# The Michigan State Dental Association

**Published:** 1873-01

**Authors:** 


### PROCEEDINGS OF SOCIETIES.

### The Michigan State Dental Association.

A preliminary meeting of this body was held on the evening of November 12th, in Jackson. George W. Stone, President of the Association, occupied the Chair.

Candidates for membership were admitted, and letters read from members who could not come.

Dr. G. E. Hays, of Buffalo, being present, was invited to take part in the deliberations.

The Association extended a similar welcome to any medical gentleman, from the city or elsewhere.

Letters were read from Dr. S. P. Cutler, of Mississippi, on the use of the microscope. Prof. Cutler claims to have discovered the ultimate molecules or atoms, which have eluded the search of scientists.

The Association then adjourned until nine o'clock on Wednesday morning.

### MORNING SESSION.

The subject of Dental Hygiene occupied the time until noon. Dr. Thomas advised dentists to urge upon parents the duty of closer attention to children's teeth. Dr. Dorrance thought that children's teeth often sustain fatal injury before they are even examined. Dr. Jackson developed the evils of bad food. Dr. Hayes urged cleanliness. Small brushes and frequent rinsings of the mouth were recommended by Dr. Hawxhurst.

### AFTERNOON SESSION.

A short paper was read by G. R. Thomas, on the subject of Dental Education, very closely summarized as follows:

Dentists should possess energy, courage, perseverance, a love of their occupation, and true gentlemany courtesy. Genius is not essential to success, but the other qualities are. He should have a strong constitution, and hence women generally ought not to enter upon the profession. He should be in love with his profession, and should be possessed with a desire for improvement in all the branches of the art. Tooth-pulling and tooth-filling are not all there is in dentistry. Dentistry begins with the study of the physiology of the human system, and he is not worthy of the name of dentist who is ignorant of dental pathology. Every dentist should be a physician; he should be able to diagnose a case as accurately as any physician might do it. In administering anæsthetics a high degree of medical judgment is necessary. The dentist should be orderly in his room and neat in his person. His rooms should be kept neat; many a dentist yearly expends more for tobacco and liquor than would suffice to put his place of business in attractive shape. A dentist ought not to use tobacco; and the public ought not to trust a dentist who uses liquor. The paper then discussed quite minutely the practice of dental colleges in issuing degrees, urging a higher standard than some institutions have insisted upon.

After much discussion, there was a general agreement upon the desirability of a dental department in our State University.

Drs. Hawxhurst, Robinson, Thomas, and several others, were appointed as a committee to confer with the Regents of the University, relative to establishing a dental department in connection with that institution.

### EVENING SESSION.

Dr. Hawxhurst, of Battle Creek, then began the reading of an interesting paper on Dental Chemistry, particularly with reference to the use of phosphates as a remedy for a scanty supply of the osseous elements in food. These substances have been prescribed by many dentists, to correct the deficiency of bone forming substances in the system. Dr. Hawxhurst gave an analysis of the tooth, showing that all the constituents of dentine are to be found richly in both vegetable and animal food. He then discussed the necessity of such mineral salts in the food, showing that their withdrawal causes enfeeblement, and ultimate death. Applying the statement to dentistry he argued that defective teeth resulted from defective organization; and that defective organization resulted, in part, from the lack of mineral salts in the food; and this led him to criticise the mill system of the country, whereby all the tooth-nourishing substances are "bolted out" of the grain, rendering it entirely inadequate to the development of sound dental organs. From the expressed opinions of leading practitioners in this and other countries, he was led to infer a widespread and profound conviction:

1. That defective organization of the teeth is the great predisposing cause of decay.

2. That defective organization arises from defective nutrition, and this largely from a scanty supply of lime salts in the ingested food.

"Millions of children in the United States fail to supply their blood with mineral salts in quantities sufficient to support normal nutrition. As a consequence their teeth are so poorly organized as not to resist the most feeble causes of decay. Nutrition has not been wholly arrested, as in the animals experimented upon by Miline Edwards, but it has been subjected to a long continued disturbance, which, though permitting a sort of stunted development of the teeth, has nevertheless entailed upon them serious molecular defects." The result of this is that the teeth of most children are unfitted to resist even ordinary causes of decay.

This portion of the paper exhibited so much thoroughness of research and boldness of statement as to provoke a long and animated discussion.

MORNING SESSION.

On Thursday morning Dr. Hawxhurst resumed the reading of his paper on "Dental Chemistry." He began by stating the question to be: How to supply the deficient elements of nutrition and furnish them to the impoverished blood and starved tissues in assimilable form. The opinion has been held that artificial lime salts, unvitalized by the plant, can not be assimulated, some even pronouncing them worthless; while others believe in their assimulation. But the proofs on either side seem to be wanting. He considered the subject one of great intricacy, and urged the rejection of all mere authority, unsupported by fact and experiment, or the most direct and rational inference from these.

He first considered the solubility of the chemically prepared phosphates in the gastric juice.

After an extended investigation, he answered this question in the affirmative, though thinking the solubility rather difficult. Having shown that they might thus be absorbed into the blood, he next asked: "Can artificial preparations of the phosphates be assimilated by the tissues? Or, will the atoms circulate as foreign substance in the blood, and finally be excreted from the system?" He showed by cautious appeal to facts, and by numerous references to European experimenters, that those practitioners are mistaken who maintain that chemically prepared phosphates are not assimilable; and who hold that none but mineral substances, that have been organized in the plant, can become a part of animal tissues. He concluded this portion of the paper, in the words of the German Wibner: "The discriminating exhibition of the phosphate of lime increases incontestably the presence of the calcarious salts in the bones" and other tissues. For easy digestibility, he preferred the lacto-phosphate, as prepared by Dusart and Blanch.

Dr. Spellman suggested that the essayist be continued as chairman of the same committee another year. He thought that those who held with the essayist, that even common articles of food are indigestible for some stomachs, phosphate of lime may act differently on different persons, had some grounds for their opinion. He thought that the views put forward were susceptible of demonstration, but held that, as the essayist had already indicated, the subject needs much investigation.

Dr. Main rendered his thanks to the Association for the instruction and entertainment afforded by the essay. He agreed with the conclusions arrived at by the essayist.

Dr. Jackson gave an interesting case where the phosphates had caused the eruption of the teeth.

Dr. E. S. Holmes thought that the eruption of the teeth was not necessarily due to the action of the phosphates. He proposed a series of experiments on pigs to test whether the phosphates can be assimilated or not.

Dr. Douglas thinks *animals* may assimilate phosphates, but not *men.*

Dr. E. S. Holmes described a case of arrested dental development in a child of eight months. On changing her diet to Graham mush her teeth were promptly erupted.

Dr. Jackson described a case of a child twenty-six months old. Its food had been farine. He gave doses of two grains of phosphate of lime with Graham diet; twelve teeth were erupted in two months.

Dr. Douglas inquired whether inorganic matter can be assimilated. He thinks not.

Dr. Main says that there seemed to be little ground for doubting Dr. Hawxhurst's conclusion. In theory we can hardly doubt it. The experience of every physician in the use of iron confirms it.

The paper and the discussion occupied the whole of the morning session, and created a great deal of interest in the minds of those present.

AFTERNOON SESSION.

Dr. Bannister, of Kalamazoo, presented a carefully prepared paper on the working of steel, particularly as applicable to perfecting the points of dental instruments. His essay contains an epitome of all the important principles and facts bearing upon the subject. Methods of beveling curved edges by means of grooved stone wheels were described and illustrated by actual demonstrations. A number of instruments were made, tempered, and beveled. Dr. Bannister by special request repeated his demonstrations next day.

EVENING SESSION.

Dr. Hawxhurst finished the reading of what the *Free Press* calls his "long and elaborate paper."

The Association then proceeded to make choice of the time and place for holding the next annual convention. It was finally decided to meet in Detroit on the second Tuesday in October.

The following officers of the Association were then elected: President, G. R. Thomas, of Detroit; Vice President, B. Bannister, of Kalamazoo; Secretary, T. B. Perry, of Grand Rapids; Treasurer, E. S. Holmes, of Grand Rapids.

Dr. Hawxhurst, of Battle Creek, was chosen a member of the Board of Censors, in place of Dr. Thomas, whose term has expired.

The fellowing gentlemen were chosen delegates to the American Dental Association, which meets at Put-in-Bay, Ohio; C. C. Jenkins, Ann Arbor; D. C. Hawxhurst, of Battle Creek; L. Douglass, Romeo; C. W. Stone, Albion.

Dr. Hunter moved that Dr. Hawxhurst be requested to prepare for publication and publish in book form the valuable paper read by him before the Association, which was unanimously adopted.

A letter was read from Prof. Taft, stating that sickness and other unfavorable contingencies had prevented his meeting with the Association.

A letter was read from Dr. E. J. Waye, in behalf of a union between the Northern Ohio Dental Association and the dentists of Michigan.

The Association voted unanimously in favor of such a union, and by resolution expressed itself in favor of going in a body to the Put-in-Bay meeting, in June, 1873.

